# Complex strabismus: a case report of hypoplasia of the third cranial nerve with an unusual clinical presentation

**DOI:** 10.5935/0004-2749.20200083

**Published:** 2024-02-11

**Authors:** Isabelle Luanna Gonçalves Tavares, Fernanda Cruz Furtado Arruda, Maria Lúcia Simão, Rosália Antunes-Foschini

**Affiliations:** 1 Hospital das Clínicas, Escola de Medicina de Ribeirão Preto, Universidade de São Paulo, Ribeirão Preto, SP, Brazil; 2 Departamento de Oftalmologia, Otorrinolaringologia e Cirurgia de Cabeça e Pescoço da Escola de Medicina de Ribeirão Preto, Universidade de São Paulo, Ribeirão Preto, SP, Brazil

**Keywords:** Oculomotor muscles/Innervation, Cranial nerve diseases, Oculomotor nerve, Strabismus, Ophthalmoplegia, Ca se reports, Músculos oculomotores/inervação, Doenças dos nervos cranianos, Nervo oculomotor, Estrabismo, Oftalmoplegia, Relatos de casos

## Abstract

Congenital cranial dysinnervation disorders are a group of complex strabismus syndromes that present as congenital and non-progressive ophthalmoplegia. The genetic defects are associated with aberrant axonal targeting onto the motoneurons, development of motoneurons, and axonal targeting onto the extraocular muscles. We describe here the surgical management of a 16-year-old boy who presented with complex strabismus secondary to hypoplasia of the third cranial nerve and aberrant innervation of the upper ipsilateral eyelid.

## Abstract (pt)

Os distúrbios de inervação craniana congênita en globam um grupo de síndromes associadas a estrabismos complexos, que se apresentam como oftalmoplegia congênita e não progressiva e são frequentemente herdadas. Os defeitos dos genes estão associados a erros no desenvolvimento ou direcionamento axonal dos motoneurônios, e erros no direc ionamento axonal para os músculos extraoculares. Este caso descreve o caso de um menino que apresenta estrabismo complexo secundário à hipoplasia do terceiro nervo craniano e inervação aberrante da pálpebra superior ipsilateral, bem como o resultado após a correção cirúrgica.

## INTRODUCTION

Congenital cranial dysinnervation disorders (CCDDs) are a group of complex strabismus syndromes which present as congenital and non-progressive ophthalmoplegia^(1)^. The genetic defects are associated with errors in axonal targeting onto the motoneurons or the extraocular muscles and aberrant motoneuron development. We describe here the surgical management in a 16-year-old boy who presented with complex strabismus involving the third cranial nerve hypoplasia and aberrant innervation of the upper ipsilateral eyelid.

## CASE REPORT

**Figure 1:**
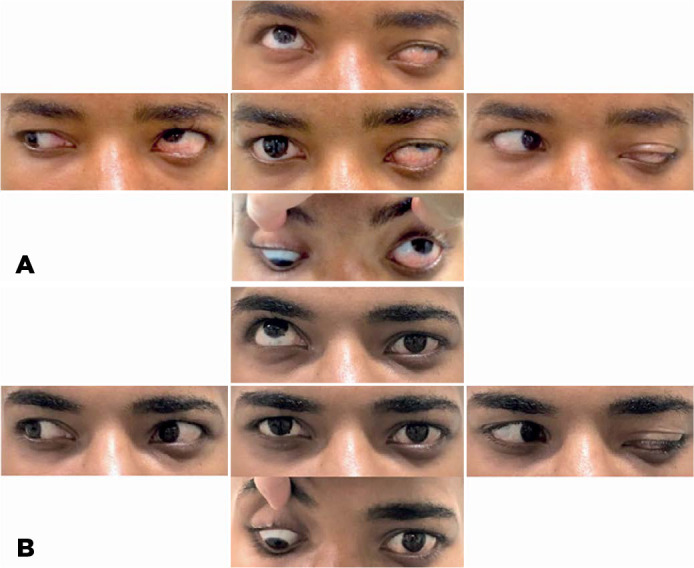
A) Pre-surgical examination: left eye (LE) hypertropia and exotropia in the primary position of gaze, a limitation in LE adduction associated with left superior eyelid opening in dextroversion, and normal LE abduction associated with the left superior eyelid closure in levo- version. B) Post-surgical results: realigned eyes in the primary position of gaze. The LE remained without elevation or depression but showed partial improvement in adduction in dextroversion. The anomalous eyelid movements remained unchanged.

A 16-year-old boy, from Ribeirão Preto, Brazil, presented with left eye (LE) hypertropia and low vision since birth. At examination, his corrected distance visual acuity was 1.0 in the right eye (RE) and 0.1 in the LE. His refraction under cycloplegia was +0.50 sph D @-1.00 cyl D in the RE and -2.50 sph D in the LE. The direct and consensual pupillary reflexes were poorly reactive to light in the LE associated with mild anisocoria (smaller pupil in the LE). In the primary position of gaze, the Krimsky test showed LE hypertropia of 50^∆^ and exotropia of 15^∆^. In dextroversion, there was a limitation in LE adduction associated with left superior eyelid opening, while in levoversion, the LE abducted normally in association with the left superior eyelid closure (Figure 1A). His LE blepharoptosis could be observed when he looked upwards, as the left superior eyelid was unable to lift normally as did the right su perior eyelid. When looking downwards, the LE remained fixed in supraduction. Biomicroscopy and fundoscopy were unreliable, and he had no relevant past medical history or family history.

**Figure 2:**
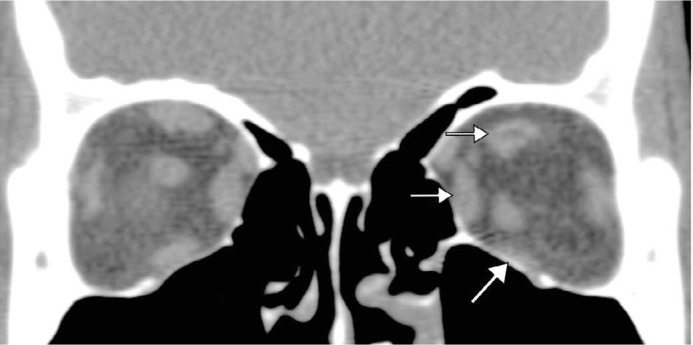
Computerized tomography (CT) showed marked left inferior rectus (LIR) and mild left superior and medial rectus muscle atrophy, with tissue lipo-substitution in the region of the left superior rectus (LSR) muscle.

**Figure 3:**
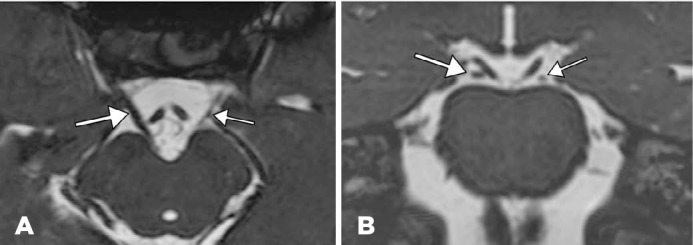
Magnetic resonance imaging (MRI) revealed hypoplasia of the third cranial nerve (CN III) with decreased thickness compared to the contralateral side.

Multi-slice computerized tomography (Figure 2) was notable for marked left inferior rectus (LIR) and mild left superior and medial rectus muscle atrophy, with significant lipo-substitution detected in the region of the left superior rectus (LSR) muscle. Nuclear magnetic resonance imaging (MRI; Figure 3, 3 Tesla, high-resolution T2 sequence) demonstrated the hypoplasia of the third cranial nerve (CN III) which displayed decreased thickness compared to that on the contralateral side. The trochlear nerves were not adequately visualized. The other cranial nerves were of normal size.

Under general anesthesia, we observed significant LSR contracture; the LE could not move downwards even with passive duction. We observed diminished thickness and width of both the LSR and LIR. We performed a 12 mm LSR recession and an 8 mm resection of the LIR, both associated with nasal transposition of their respective insertions. After surgery, there was a substantial improvement in the LE hypertropia. However, the Krimsky test still revealed a LE exotropia of 20^∆^ in the primary position of gaze. Two months later the patient underwent a second surgery, in which we performed a 10 mm recession of the right lateral rectus. After surgery, there was a clear and substantial improvement in the primary position of gaze. The LE remained without elevation or depression. However, there was a partial improvement in adduction. The anomalous eyelid movements remained unchanged (Figure 1B).

## DISCUSSION

The combination of the clinical presentation, the MRI and computerized tomography imaging led us to the diagnosis of complex strabismus due to a congenital cranial dysinnervation disorder (CCDD)^(2-4)^. CCDDs are a group of complex strabismus syndromes which present as congenital and non-progressive ophthalmoplegia^(1)^. They are often inherited, and the genetic defects are asso ciated with errors in axonal targeting and motoneuron development^(4-6)^. Consequently, incomitant extraocular motility abnormalities are observed; some of these are due to the absence of motoneurons and others are secondary to aberrant innervation of the extraocular muscles. When defects in ocular motility are mainly vertical, there are likely to be secondary to aberrancies in cranial nerves III or IV. When the defects of ocular motility are predominantly horizontal, there is likely to be an aberrancy related to cranial nerves III or VI. The CCDDs include congenital ptosis as well as Duane, Möbius and Marcus Gunn syndromes. In this patient, however, the clinical findings did not fit precisely with any of the previously described presentations of CCDD. The absence or decrease of specific LE movements (decreased adduction, no infraduction or supraduction, and LE blepharoptosis associated with limited superior eyelid lifting) are compatible with hypoplasia of the third cranial nerve. This was observed in the neuroimaging, as well as in the CT scan that revealed decreased thickness of the extraocular muscles. Third nerve hypoplasia has been described as congenital fibrosis of the extraocular muscles (CFEOM) types 1, 2, and 3^(1)^; strabismus fixus in supraduction is not commonly associated with this syndrome. The patient also had a left-sided small and poorly reactive pupil. Poorly reactive and miotic pupils have been described in patients with CFEOM type 2^(7,8)^; this finding results from mutations in the gene PHOX2A and are secondary to dysinnervation of targets of the third and fourth cranial nerves. The abnormal closure of the left superior eyelid in levoversion led us to hypothesize that aberrant innervation from motorneurons from the left abducens nerve might be innervating the orbicular muscle (which is ordinarily innervated by the seventh cranial nerve), leading to eyelid closure when the patient looks to the left (abducts) and eyelid opening when he looks to the right, because the left lateral rectus is not innervated. The upper lid retraction in adduction has also been reported in association with aberrant third nerve regeneration^(9)^. However, aberrant oculomotor regeneration is typically associated with previous ocular trauma and one would not expect to see extraocular muscle atrophy, as observed in the LIR, LSR and left medial rectus muscles.

In conclusion, this case describes a rare form of congenital strabismus associated with hypoplasia of the third cranial nerve, pupillary involvement and aberrant innervation of the left superior eyelid. Complex strabismus associated with a cranial dysinnervation disorder presents a major challenge both in terms of diagnosis and treatment. A detailed examination of ocular motility, ophthalmologic alterations and neuroimaging are fundamental in the appropriate evaluation and treatment of these patients.
